# A cisplatin conjugate with tumor cell specificity exhibits antitumor effects in renal cancer models

**DOI:** 10.1186/s12885-023-10878-3

**Published:** 2023-06-02

**Authors:** Stefan Mrdenovic, Yanping Wang, Lijuan Yin, Gina Chia-Yi Chu, Yan Ou, Michael S. Lewis, Marija Heffer, Edwin M. Posadas, Haiyen E. Zhau, Leland W. K. Chung, Mouad Edderkaoui, Stephen J. Pandol, Ruoxiang Wang, Yi Zhang

**Affiliations:** 1 Division of Hematology, Department of Internal Medicine, University Hospital Osijek, Osijek, Croatia; 2 Department of Medicine, Cedars-Sinai Medical Center, Los Angeles, CA USA; 3 Department of Surgery, Cedars-Sinai Medical Center, Los Angeles, CA USA; 4 Departments of Pathology, Cedars-Sinai Medical Center and the VA Greater Los Angeles Healthcare System, Los Angeles, CA USA; 5 Biomedical Imaging Research Institute, Department of Biomedical Sciences, Cedars-Sinai Medical Center, 8700 Beverly Boulevard, Davis 3059, 90048 Los Angeles, CA USA; 6 Department of Internal Medicine, Family Medicine and History of Medicine, Faculty of Medicine, J. J. Strossmayer University of Osijek, Osijek, Croatia; 7 Department of Medical Biology and Genetics, Faculty of Medicine, J. J. Strossmayer University of Osijek, Osijek, Croatia

**Keywords:** Kidney cancer, Heptamethine carbocyanine dye, Cisplatin, Conjugate, Cell death

## Abstract

**Background:**

Clear cell renal cell carcinoma (ccRCC) is the most common type of kidney cancer and is notorious for its resistance to both chemotherapy and small-molecule inhibitor targeted therapies. Subcellular targeted cancer therapy may thwart the resistance to produce a substantial effect.

**Methods:**

We tested whether the resistance can be circumvented by subcellular targeted cancer therapy with DZ-CIS, which is a chemical conjugate of the tumor-cell specific heptamethine carbocyanine dye (HMCD) with cisplatin (CIS), a chemotherapeutic drug with limited use in ccRCC treatment because of frequent renal toxicity.

**Results:**

DZ-CIS displayed cytocidal effects on Caki-1, 786-O, ACHN, and SN12C human ccRCC cell lines and mouse Renca cells in a dose-dependent manner and inhibited ACHN and Renca tumor formation in experimental mouse models. Noticeably, in tumor-bearing mice, repeated DZ-CIS use did not cause renal toxicity, in contrast to the CIS-treated control animals. In ccRCC tumors, DZ-CIS treatment inhibited proliferation markers but induced cell death marker levels. In addition, DZ-CIS at half maximal inhibitory concentration (IC50) sensitized Caki-1 cells to small-molecule mTOR inhibitors. Mechanistically, DZ-CIS selectively accumulated in ccRCC cells’ subcellular organelles, where it damages the structure and function of mitochondria, leading to cytochrome C release, caspase activation, and apoptotic cancer cell death.

**Conclusions:**

Results from this study strongly suggest DZ-CIS be tested as a safe and effective subcellular targeted cancer therapy.

**Supplementary Information:**

The online version contains supplementary material available at 10.1186/s12885-023-10878-3.

## Background

Renal cell carcinoma (RCC) constitutes more than 3% of global human cancer diagnoses and is often a metastatic disease with a belligerent course [1–3]. Approximately 75% of RCCs have a histologically clear cell (ccRCC) morphology, characterized by von Hippel-Lindau (VHL) gene mutations or its promoter hypermethylation [4]. Most RCC cases are highly aggressive, showing a bleak 5-year survival rate of 12% and causing more than 175,000 worldwide deaths per year [5].

The treatment of ccRCC has evolved rapidly in recent years. For localized tumors, surgery is the preferred option. For metastatic or relapsed diseases, systemic therapy is the choice [6, 7]. Many of the treatment strategies are based on multi-targeted receptor tyrosine kinase inhibitors or immunotherapy-based combinatory regimens [6]. These strategies are met with significant therapeutic resistance, with median overall survival of fewer than 48 months [8]. Clinical trials with single agents, combinatory chemotherapy, and metronomic or circadian infusion therapy with agents including vinca alkaloids, gemcitabine, and fluoropyrimidine derivates showed only modest results [9]. The mechanism of resistance in ccRCC is complex, as tumor cell heterogeneity, epithelial to mesenchymal transition, bypass pathway activation, lysosomal drug sequestration, noncoding RNA function, immune escape, and the tumor microenvironment modulation may all support enhanced tumor cell survival [10]. In recent years, subcellular targeted cancer therapy [11, 12] has been tested to treat cancers resistant to conventional therapies. In this regard, an ideal agent for the new therapy should have both tumor-cell specificity and tumor cell killing activity to produce a substantial effect.

We have previously identified a group of near-infrared (NIR) heptamethine carbocyanine dyes (HMCDs) that possess tumor imaging and tumor homing properties [13]. Cancer specific accumulation of HMCDs is mediated in part by organic anion-transporting polypeptides (OATPs), which facilitates a preferential uptake in tumor but not normal cells as demonstrated in mouse and dog tumor models, patient-derived xenografts and perfused kidney tumor specimens from patients [14–18]. We demonstrated that one of the HMCDs, referred to as DZ, could be used as a targeting vehicle to deliver therapeutic payloads specifically to tumor cells. In one of these studies, we developed DZ-CIS by conjugating DZ with cisplatin (CIS), which is a widely used chemotherapeutic agent [19–21] but is notorious for its severe side effects, especially on the structure and function of the kidneys [22, 23]. The DZ-CIS is a NIR fluorescent compound that can effectively kill cisplatin-resistant Burkitt’s lymphoma cells by attacking mitochondrial and lysosomal structure and function [24]. DZ-CIS is thus a typical antitumor agent for subcellular targeted cancer therapy. With tumor cell specificity, this novel agent can inhibit xenograft tumor formation without causing detectable side effects.

In this study, we assessed the effect of the DZ-CIS conjugate on ccRCC in comparison to the CIS. The DZ-CIS conjugate, with potent tumor cell killing activity and absence of renal toxicity, is shown as an ideal new candidate for RCC treatment.

## Methods

### Reagents and characterization

High quality chemicals and reagents were purchased from standard sources such as Sigma-Aldrich (St. Louis, MO, USA) and VWR International (Radnor, PA, USA). Deionized water (18.2 Ω) used for making solutions was obtained from Milli-Q Direct Ultrapure Water System from Millipore (Billerica, MA, USA). All intermediates were characterized by proton nuclear magnetic resonance (^1^H NMR) and mass spectrometry (MS) analysis, and the purity of compounds was analyzed by high-performance liquid chromatography (HPLC). ^1^H NMR data were collected on a Bruker 400 MHz spectrometer (Bruker, Billerica, MA) using standard parameters, while chemical shifts are reported in ppm (δ) in reference to residual non-deuterated solvent. Electrospray ionization (ESI) MS analysis was performed on new compounds with an LTQ Orbitrap Elite system (ThermoFisher Scientific, Waltham, MA, USA) at the Mass Spectrometry and Biomarker Discovery Core facility of the Cedars-Sinai Medical Center, Los Angeles, CA.

### Synthesis of the DZ-CIS conjugate

Detailed protocol for DZ-CIS synthesis has been reported [24]. In brief, CIS was oxidized with hydrogen peroxide to form *cis*, *cis*, *trans*-diaminedichloro-dihydroxyplatinum (IV) (oxoplatin) according to the reported method [25]. DZ (500 mg, 0.71 mmol) was added to a suspension of the oxoplatin compound (350 mg, 1.05 mmol) in dimethyl sulfoxide (DMSO, 20 mL), followed by 1-ethyl-3-(3-dimethyllaminopropyl) carbodiimide hydrochloride (204 mg, 1.06 mmol) and dimethylaminopyridine (20 mg, 0.16 mmol) and the mixture was stirred for 20 h at room temperature. The product was purified with C18 reversed-phase (RP) silica chromatography and eluted with methanol-water to afford DZ-CIS as a dark green solid.

### Cell culture

Human ccRCC cell lines of Caki-1, 786-O, ACHN, and SN12C were obtained from American Type Culture Collection (ATCC, Manassas, VA). The source of immortalized human embryonic kidney HEK293 cells was purchased from ATCC [13]. Mouse Renca cells were provided by Dr. Hyung L. Kim of the Department of Surgery of the Cedars-Sinai Medical Center. Human primary renal epithelial cell culture was obtained from *ex vivo* culture of a ccRCC surgical tumor specimen with approval by the Institutional Review Board (IRB No. Pro00031870). All methods were carried out in accordance with the IRB guidelines and regulations. Informed consent was obtained from the subject. Primary cells at passages 4 and 5 were used. Cells were cultured in RPMI 1640 medium (Life Technologies, Carlsbad, CA) supplemented with 10% fetal bovine serum (Atlanta Biologicals, Flowery Branch, GA), 100 IU/ml penicillin, and 100 µg/ml streptomycin (ThermoFisher Scientific) at 37°C in a humidified incubator supplemented with 5% CO_2_.

### Cell proliferation assay

Cells (5 × 10^3^/well) of quadruplet wells in 96-well plates (USA Scientific, Irvine, CA) were exposed to CIS (Selleck Chemicals, Houston, TX) or DZ-CIS for 24 h, with the final concentration of solvent DMSO (Sigma-Aldrich) never exceeding 1%. Cells were then stained with 10% 3-(4,5-Dimethyl-2-thiazolyl)-2,5-diphenyl-2 H-tetrazolium bromide (MTT, Sigma-Aldrich) for 4 h and decolorized by adding 100 µl of acidic 2-propanol. The extinction of supernatant was read at an absorbance maximum of 595 nm using a microplate reader (Bio-Rad Laboratories, Hercules, CA). For drug synergism testing, human cancer cells were exposed to exponentially increasing concentrations (0 µM to 64 µM) of everolimus or temsirolimus (Selleck Chemicals, Houston, TX) plus 4 µM DZ-CIS for 24 h. The coefficient of drug interaction (CDI) was calculated based on the Combination Index Theorem by Chou T.C. and Talalay P, using a formula CDI = AB/(A + B) where CDI < 1 indicates synergism, CDI = 1 indicates additivity, and CDI > 1 indicates antagonism [26].

### Apoptosis assay and western blotting

For the caspase activity assay, cells treated with DZ-CIS for 24 h were measured for caspase 3/7 enzymatic activities by the Caspase-Glo® 3/7 Assay System (Promega, Madison, WI) with the recommended protocol by the manufacturer. Luminescence intensity was acquired using a LUMIstar Omega microplate luminometer (BMG Labtech, BioTek, Winooski, VT). For western blot analysis, our previously reported protocol was used [24]. Antibodies to poly ADP-ribose polymerase (PARP), caspase 3, and caspase 9 were from Cell Signaling Technology (Danvers, MA). Antibodies to β-actin were purchased from Santa Cruz Biotechnology (Dallas, TX). Horseradish peroxidase (HRP)-conjugated secondary antibodies (Santa Cruz Biotechnology) were used.

### Fluorescence microscopy

Cells cultured in chamber slides (Nalge Nunc International, Rochester, NY) were stained with 4 µM DZ-CIS. After removing the staining medium, the cells were washed in phosphate buffered saline (PBS) 3 times. The slide was then counterstained with Hoechst 33342 (1 µg/ml, ThermoFisher Scientific) for 10 min, fixed in 4% paraformaldehyde (Sigma-Aldrich), and subjected to analysis of NIR fluorescence dye uptake with an Eclipse Ti-E confocal microscope (Nikon Corporation, Tokyo).

### Tumor implant study

Male 4- to 6-week-old NCr nude (*Foxn1*^*Nu*^*/Foxn1*^*Nu*^) mice (n = 10) and BALB/cJ mice (n = 15) purchased from the Jackson Laboratory (Bar Harbor, ME) were subjected to subcutaneous (*s.c*) tumor inoculation, for which 5 × 10^6^ ACHN cells or 2 × 10^5^ Renca cells in 50 µl of 50% Matrigel© (Corning, Corning, NY) were injected to each flank, producing 2 inoculations per mouse. The mice were then randomized into three groups and were treated intraperitoneally (*i.p*) twice a week with 10 mg/kg of DZ-CIS in 100 µl of PBS consisting of 5% DMSO and 15% PEG-40 (Sigma-Aldrich). The study approval was obtained from the Institutional Animal Care and Use Committee (IACUC), Cedars-Sinai Medical Center. Tumor growth was monitored by measuring the tumor dimensions with a caliper, and tumor volume was calculated using the formula: tumor volume = (length × width^2^)/2. The endpoint of the study was set as statistically significant tumor suppression in the treatment group. At the end of the studies *s.c* tumors, livers, and kidneys were excised, fixed in 4% paraformaldehyde, and embedded in paraffin for histopathological analyses.

### NIR fluorescence tumor imaging

Animals were subjected to imaging 72 h after the final DZ-CIS *i.p* injection, with an IVIS Lumina XR Imaging System (PerkinElmer, Waltham, MA) equipped with fluorescent filter sets (excitation/emission, 745/820 nm), with automatic background fluorescence subtraction.

### Immunohistochemical analysis (IHC)

IHC analysis was performed as we previously reported [27]. Primary antibodies against the cleaved caspase 3 (Cell Signaling Technology), PECAM-1 (CD31, Novus Biologicals, Centennial, CO), SOX2 and Ki67 (Santa Crus Biotechnology, Dallas, TX), and the M30 epitope of soluble caspase-cleaved keratin 18 (Sigma-Aldrich) were used. Image acquisition was performed using a digital camera (Nikon Corporation).

### Statistical analysis

All results were presented as mean ± standard deviation (STDEV) obtained from at least three independent tests. The normality of distribution was assessed using the Kolmogorov-Smirnov test. Parametric data were compared using Student’s *t*-test. Nonparametric data were compared using Mann-Withney U test and SPSS software version 15 (IBM Corporation, Armonk, NY). The half maximal inhibitory concentration (IC50) values were calculated using a nonlinear regression method after normalizing the acquired values using GraphPad Prism version 8.4.3 Software (GraphPad Software, La Jolla, CA) by dose-response inhibition XY nonlinear regression analysis of log(inhibitor) vs. normalized response from 0 to 100% - model equation Y = 100/(1 + 10^(X-LogIC50)).

## Results

1. The DZ-CIS conjugate inhibits ccRCC cell viability and proliferation.

**Fig. 1:**
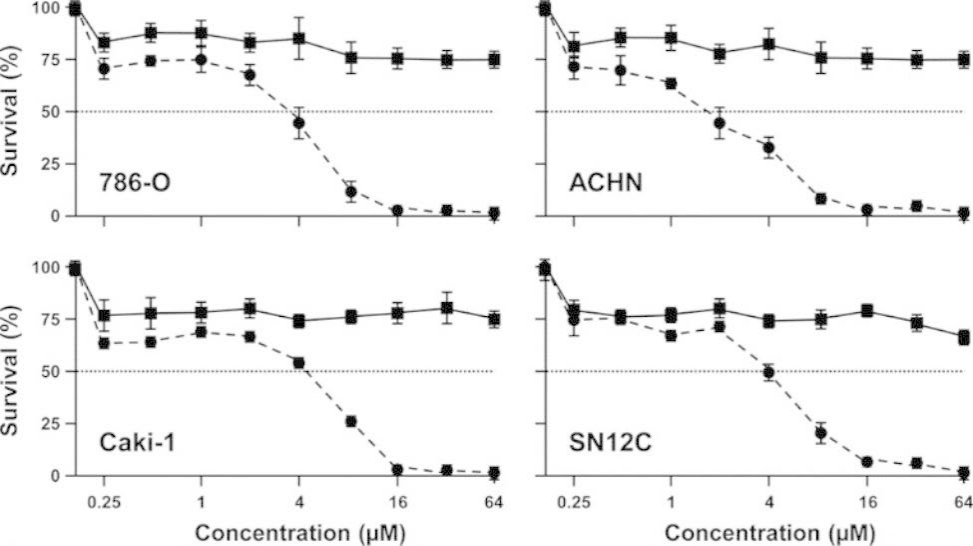
DZ-CIS kills ccRCC cells in a dose-dependent manner. The 786-O, ACHN, Caki-1, and SN12C human ccRCC cells were treated with increasing concentrations of DZ-CIS (filled circle) for 24 h, followed by MTT staining. Same concentrations of CIS (filled square) were used as control. Each data point represents the mean of a triplicate assay ± STDEV.

We have described the structural design procedures, chemical synthesis, and product characterization of the DZ-CIS in detail [24]. To investigate tumor targeting and cancer inhibition efficacy of DZ-CIS, we used ccRCC cells as subjects that harbor intrinsic resistance to cisplatin. 786-O is a ccRCC cell line isolated from a primary tumor [28], while Caki-1 is derived from a ccRCC metastatic site [29]. ACHN represents a metastatic papillary RCC cell line isolated from pleural effusion [30]. SN12C is of mixed granular and clear cell origin [31]. 786-O and SN12C cells harbor mutated p53 while Caki-1 and ACHN possess a wild type p53 [32, 33]. Cells were treated with either CIS or DZ-CIS in concentrations ranging from 0.25 to 64 µM for 24 h (Fig. 1). All treated cells were resistant to CIS, with IC50 > 64 µM (Table 1). On the other hand, there was a significant inhibitory effect by DZ-CIS treatment, with IC50 values ranging from 1.94 for ACHN to 3.71 for 786-O and Caki-1 cells (Table 1). In contrast to the cancer cells, human primary renal epithelial cells and HEK293 cells responded to the DZ-CIS treatment with a much weaker growth inhibition at 32 µM and 64 µM concentrations. These results demonstrated that DZ-CIS was a potent inhibitor of RCC cell proliferation in a dose-dependent manner.

**Table 1:** IC50 (µM) at 24 h

	CIS	DZ-CIS	Significance
786-O	> 64	3.71	p < 0.0001
ACHN	> 64	1.94	p < 0.0001
Caki-1	> 64	3.71	p < 0.0001
SN12C	> 64	3.67	p < 0.0001

2. DZ-CIS is cytocidal to RCC cells.

**Fig. 2:**
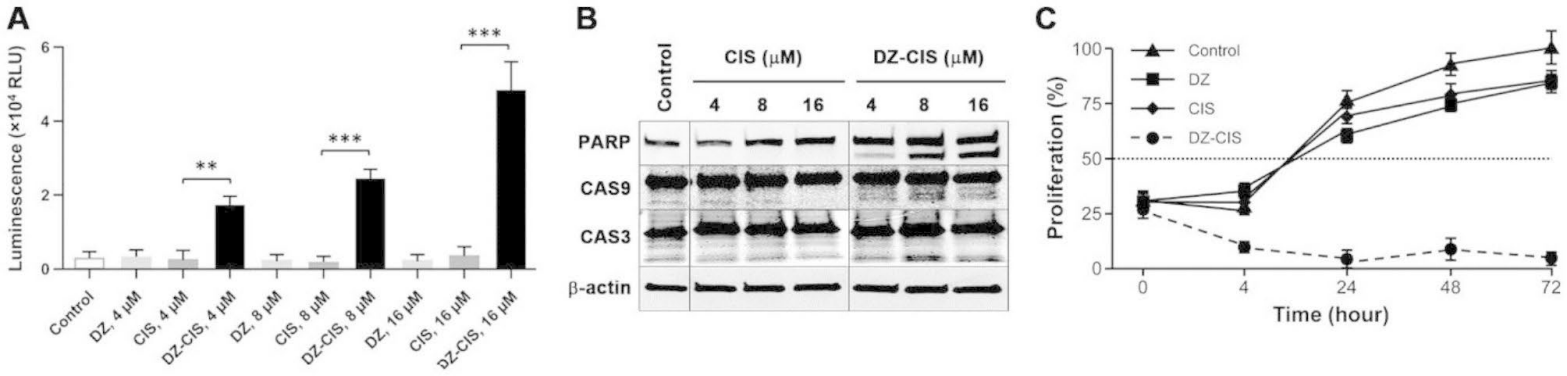
DZ-CIS is cytocidal. Representative results with Caki-1 cells are shown. **A**, DZ-CIS treatment elicits caspase 3/7 enzymatic activities as detected with the Caspase-Glo® 3/7 Assay System, 8 h into the treatment with specified DZ-CIS concentrations. Data derived from triplicate treatment are shown as average ± STDEV. **: p < 0.05; ***: p < 0.01. **B**, DZ-CIS induced caspase activation and substrate protein cleavage. Caki-1 cells treated with specified concentrations of DZ-CIS for 8 h were subjected to western blotting for apoptosis-related proteins. The study was repeated for at least once and similar results were obtained. **C**, DZ-CIS-treated cells lose viability. To distinguish cytocidal from cytostatic activity, Caki-1 cells were treated for prolonged time to determine whether the treated cells may proliferate in a slowed rate. MTT staining was used at different time points to determine proliferation. Cells treated with 8 µM DZ-CIS completely lost proliferative activity, while cells treated with control agents at the same concentration proliferated within the 72 h

Morphologic changes in treated cells suggested that DZ-CIS directly killed RCC cells, in agreement with our findings in treating lymphoma cells [24]. Under phase contrast microscope, ccRCC cells treated with different doses of CIS all remained morphologically intact, without any signs of structural disruption, even after 72 h of treatment. These observations indicate that any inhibitory effect by this agent is mostly cytostatic. On the other hand, DZ-CIS treatments resulted in widespread cell death within 24 h, accompanied by apoptotic morphologies in a dynamic process like the one observed with the lymphoma cells [24]. We carried out assays to determine the mode of DZ-CIS-induced cancer cell killing. There was a clear dose-dependent caspase activation, as detected with the increased enzymatic activity of caspases-3 and caspase-7 in RCC cells treated for 12 h by DZ-CIS in the range of 8 µM to 16 µM (Fig. 2A). These results were consistent with an enhanced cleavage of caspase-3 (CAS3), caspase-9 (CAS9), and PARP apoptotic cascade proteins, as detected by western blotting (Fig. 2B). All these results suggest that DZ-CIS is cytocidal to malignant cells by eliciting the apoptotic cascade. In addition, the cytocidal activity could be confirmed by extended cultures. In these studies, DZ-CIS completely suppressed proliferation as all the cells were killed. In contrast, cell growth under DZ or CIS treatment may become decelerated, but considerable proliferative activity persisted for 24, 48, and 72 h (Fig. 2C).

3. DZ-CIS sensitizes RCC cells to mTOR inhibitors.

**Fig. 3:**
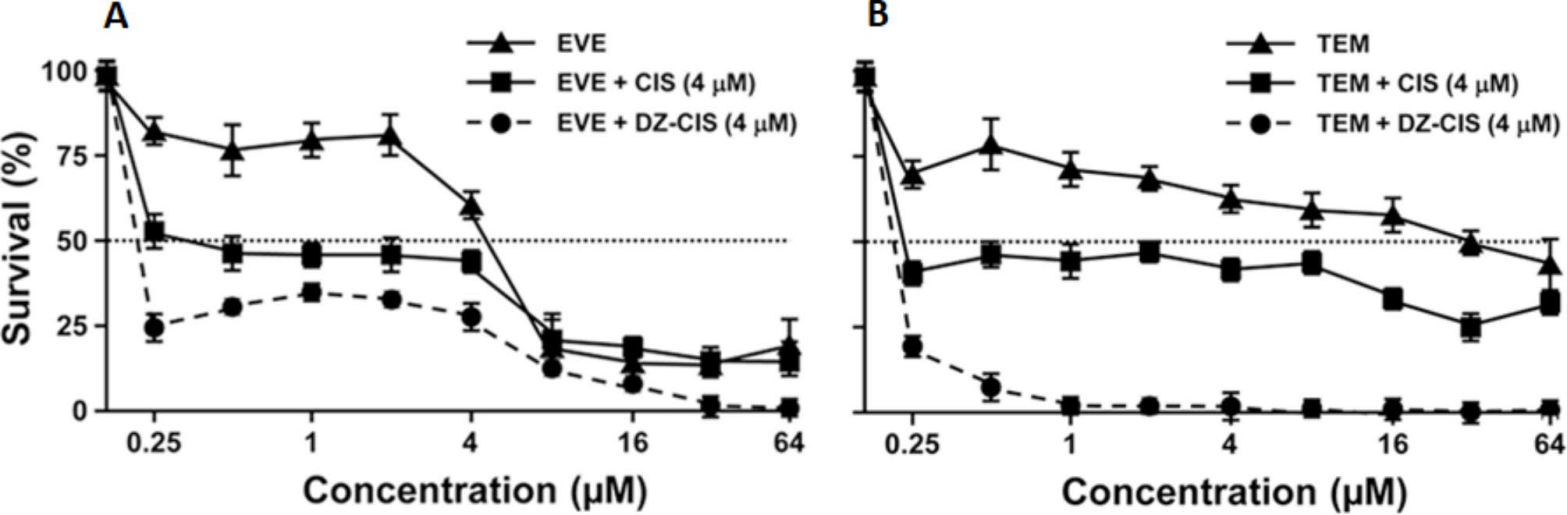
DZ-CIS may sensitize RCC cells to mTOR inhibitor treatment. Results with Caki-1 cells are presented. In these studies, Caki-1 cells were treated with increasing concentrations of mTOR inhibitors EVE (**A**) or TEM (**B**), in combination with 4 µM DZ-CIS, for 24 h followed by MTT staining. Data are shown as the average of a triplicate assay ± STDEV.

To further investigate the anti-proliferation ability, we combined DZ-CIS with mTOR inhibitors everolimus (EVE) or temsirolimus (TEM), which was used to inhibit advanced RCC in case sunitinib and/or sorafenib treatment fails [34]. RCC cells were exposed to increasing concentrations of the inhibitors ranging from 0.25 to 64 µM in combination with a reduced dose (4 µM) of CIS or DZ-CIS. Treatment with mTOR inhibitor + CIS induced a minor decrease in IC50 concentration, while DZ-CIS caused a pronounced IC50 decrease to 0.3 µM for EVE + DZ-CIS (CDI = 0.028) and 0.19 µM for TEM + DZ-CIS (CDI = 0.003) (Fig. 3A and B; Table 2). Noticeably, treatment with TEM + DZ-CIS leads to complete in vitro cell killing with 1 µM (Fig. 3B). These results encourage a possibility of a synergistic effect of DZ-CIS with mTOR inhibitors.

**Table 2:** IC50 (µM) with Caki-1 cells at 72 h

	Everolimus	Temsirolimus
	3.85	23.38
CIS (4 µM)	0.9	1.19
DZ-CIS (4 µM)	0.3	0.19

4. DZ-CIS accumulates preferentially in RCC cells over primary kidney epithelial cells.

**Fig. 4:**
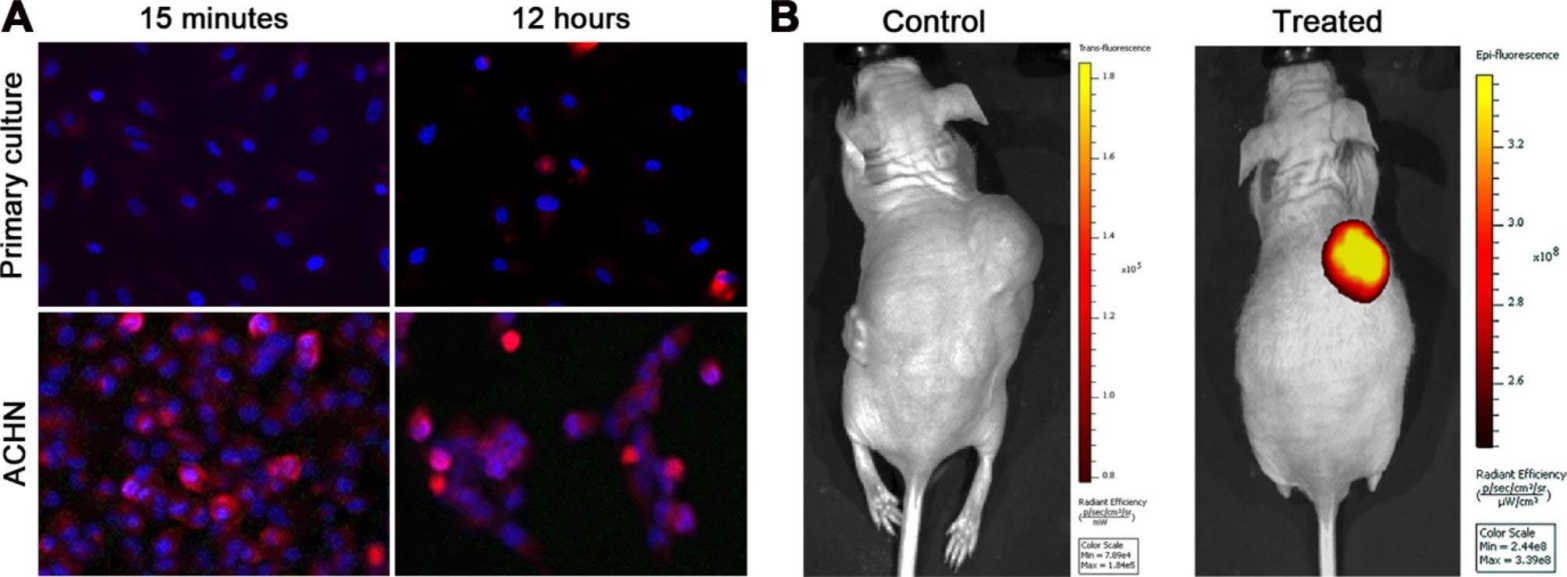
Tumor cell specificity of the DZ-CIS. Representative results are shown. **A**, ACHN cells were treated with 8 µM DZ-CIS for specified times. After removing the treatment medium and washing the cells for three times in PBS, the cells were stained with Hoechst 33342 and subjected to fluorescence microscopy (100×). A primary culture of human kidney epithelial cells (Primary culture) was used as control. Merged Hoechst 33342 and DZ-CIS images are shown. **B**, DZ-CIS preferentially accumulate in tumors. A mouse bearing an ACHN xenograft tumor was treated *i.p* with a dose of DZ-CIS (10 mg/kg), and was subjected to NIR whole body imaging, 72 h after the DZ-CIS injection. NIR whole body imaging from an untreated tumor-bearing mouse is used for comparison

Preferential accumulation of DZ-CIS in cultured RCC cells over normal kidney epithelial cells was inspected microscopically. After a 15-minute treatment with 8 µM DZ-CIS, little stain was seen in normal kidney epithelial cells. In contrast, all RCC cells were stained positively as detected based on NIR fluorescence (Fig. 4A), suggesting enhanced uptake and retention by RCC cells. On a prolonged treatment (24 h), no damage was seen to normal kidney epithelial cells, but an almost complete killing was observed in RCC cells. Under visual inspection, there was broad apoptotic nuclear fragmentation concurrent with other morphologic changes of apoptosis. To assess DZ-CIS accumulation in vivo, NCr nude mice bearing *s.c* ACHN xenograft tumors were injected with a 10 mg/kg single dose of DZ-CIS. Whole body imaging detected strong NIR images, specifically in the xenografts, with an average maximal radiant efficiency value around 3.48 × 10^8^ (n = 4) when detected 72 h after the last DZ-CIS administration. In comparison, background signals were about 1.93 × 10^5^ in any tissue or organs of the host (Fig. 4B). In repeated experiments, the accumulation of DZ-CIS in xenografts appeared persistent, as NIR signals in xenografts could be detected reproducibly within 14 days following a single DZ-CIS administration.

5. DZ-CIS inhibits RCC tumor growth in mouse models.

**Fig. 5:**
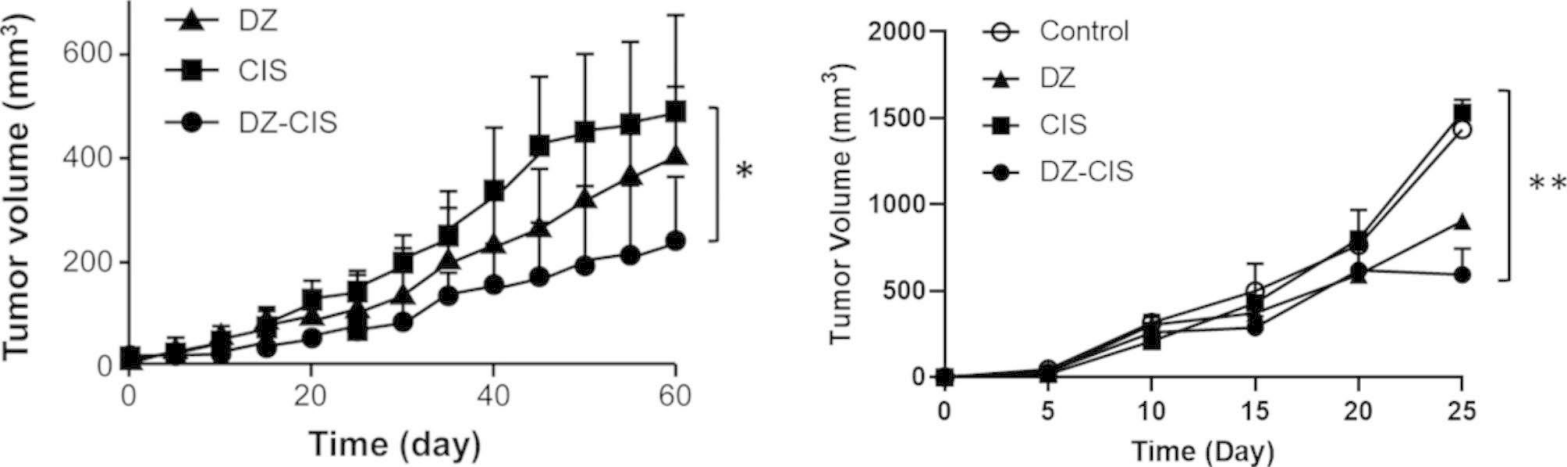
DZ-CIS inhibits RCC tumor formation in mice. Results with the ACHN xenografts (left) and the Renca allografts (right) are shown. Data are shown as average tumor volume ± STDEV. *: p < 0.05

To determine whether the growth inhibition and tumor cell killing activity observed in cultured RCC cells is reserved in vivo, we evaluated DZ-CIS in two in vivo models, human ACHN RCC *s.c* xenograft tumor formation in NCr nude mice and Renca mouse RCC allografts on BALB/cJ mice. Following treatment with DZ-CIS (10 mg/kg, *i.p*) twice a week, significant decreases in tumor volume were observed compared to CIS- or vehicle-treatment groups (p < 0.05) (Fig. 5).

6. DZ-CIS inhibits RCC tumor formation without causing renal toxicity.

**Fig. 6:**
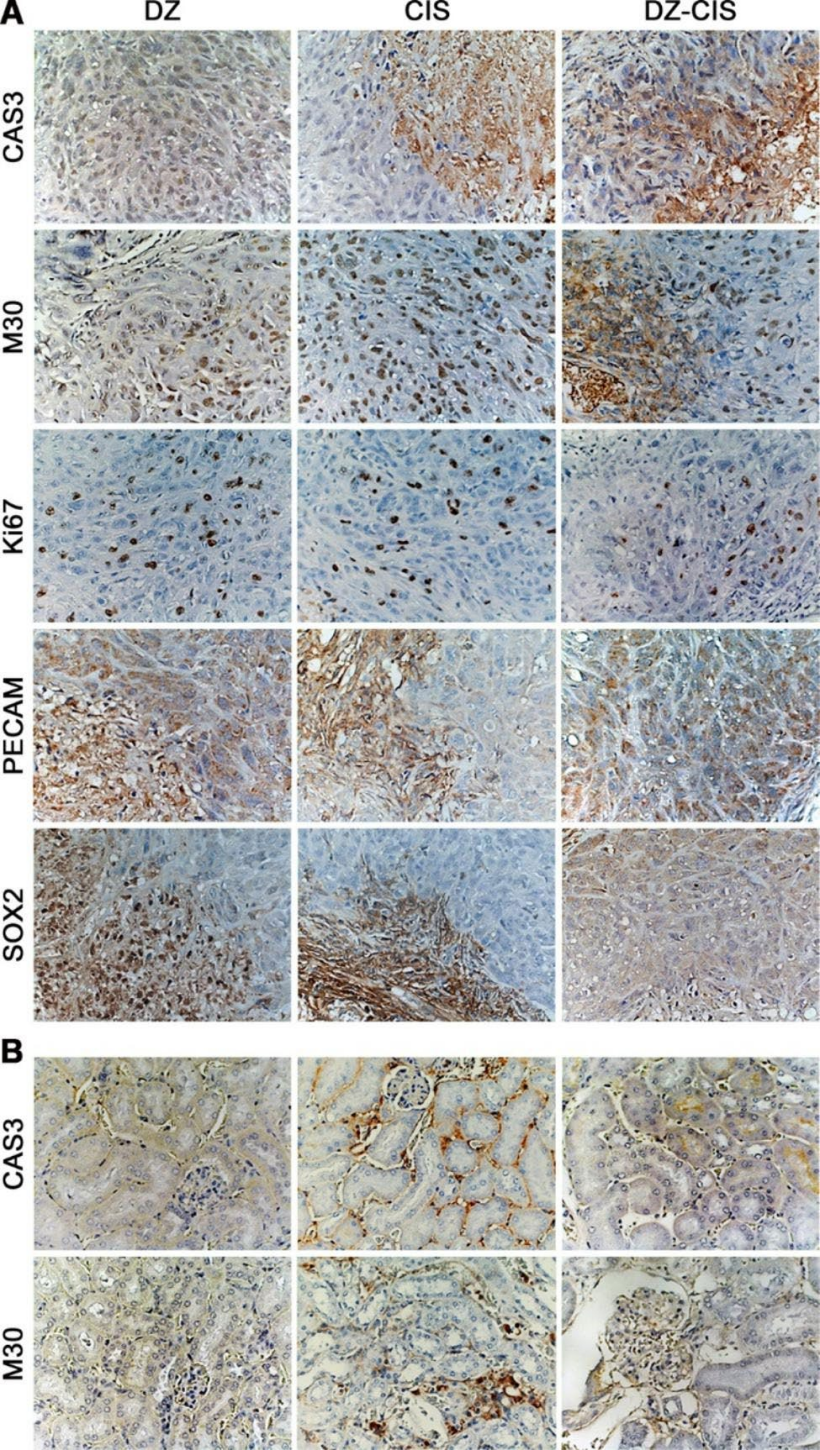
IHC results supporting tumor inhibition effect of the DZ-CIS. Representative results are shown. **A**, ACHN xenograft tumors treated with 10 mg/kg DZ-CIS for 60 days were examined for IHC changes (100×). The tumors treated with DZ or CIS from the same study were used as control. **B**, host kidneys from the tumor-bearing mice treated with 10 mg/kg DZ-CIS for 60 days were analyzed for any structural or expressional abnormalities. Kidneys from the DZ- or CIS-treated mice were used for comparison (100×)

We examined selected markers in the ACHN xenograft tumor specimens. Apoptosis markers of cleaved caspase 3 (CAS3), together with the caspase-cleaved and formalin-resistant cytokeratin 18 neo-epitope (M30), were increased in the DZ-CIS treated group compared to CIS-treated group (Fig. 6A). A decreased Ki67 stain in the DZ-CIS group may be implying a decrease in tumor cell proliferation rate. Decreased PECAM-1 suggests decreased tumor angiogenesis. SOX2 activation in renal cancer is associated with the induction of a stem cell-like phenotype [35] and is a predictor of poor prognosis in RCC [36]. Decreased SOX2 expression in the DZ-CIS treated group could be related to the decrease in the tumor stem cell population.

Since one of the major side effects of CIS chemotherapy is renal toxicity [37], we assessed DZ-CIS conjugate toxicity by detecting the appearance of apoptotic markers in the kidney of the mouse host. Mice treated with CIS were found with an increased intensity of CAS3 and M30 in kidney glomeruli and proximal tubules (Fig. 6B). In contrast, treatment with DZ-CIS resulted in almost no evidence of cleaved caspase 3 and M30 staining signal. These findings implied that different from CIS, in vivo use of the DZ-CIS conjugate had a much less toxic effect on normal mouse kidney tissue. This data confirmed earlier renal sparing properties of DZ-CIS in Burkitt’s lymphoma model [24]. Mice subjected to DZ-CIS treatment did not show any structural or expressional abnormalities in the kidneys and the liver.

## Discussion

RCC tumors have an innate tendency to develop resistance to chemotherapies and targeted therapies, posing a major obstacle to the treatment of disease progression and tumor metastasis [10, 38, 39]. Rather than killing tumor cells, most chemotherapeutics act on the mechanism of cell division to slow down growth rate of the proliferative cells, without sizable effects on senescent or growth arrested cells. Serving for such critical cellular functions as growth and proliferation, the cell division machinery is known to be protected by multiple strategies, which can be hijacked by cancer cells for chemotherapeutic resistance. On the other hand, as small-molecule inhibitors used in targeted therapies may block specific signal transductions, many signal transductions are not vital to cell survival, partly because signal transductions can take parallel pathways, which can also be adopted by tumor cells for therapeutic resistance. Tumor cells may also possess exceptional survival capability, making RCC one of the most insensitive malignancies to conventional chemotherapies or small-molecule targeted treatments.

Subcellular targeted cancer therapy may be an alternative strategy for effectively controlling RCC progression and metastasis [11, 12], and the DZ-CIS conjugate may be an exemplary compound for this purpose. Our results so far demonstrate that DZ-CIS is cytocidal, capable of either killing ccRCC cells by itself (Figs. 1, 2 and 6A) or enabling small-molecule inhibitors to kill (Figs. 3 and 5). Mechanistically, the effect of DZ-CIS is shown to be on subcellular organelles, where it is seen to destruct mitochondrial structural integrity and oxidative phosphorylation leading to cytochrome C leakage and caspase activation [24]. At the same time, DZ-CIS has tumor-cell specificity, being preferentially taken up and retained by malignant cells over normal cells (Fig. 4). Though the mechanism of tumor-cell specificity remains to be conclusively illustrated, tumor cell killing activity and tumor cell targeting specificity make DZ-CIS a promising agent for the clinical treatment of RCC, since DZ-CIS-mediated subcellular targeted cancer therapy can circumvent RCC’s therapeutic resistance.

We have previously identified a group of NIR heptamethine carbocyanine dyes (DZ) that possess tumor imaging and tumor homing properties [13]. Its properties are to selectively accumulate inside tumor cells without the possibility of fast or effective efflux. On the other hand, there is evidence of its accumulation in mitochondria and lysosomes [24]. We have previously successfully conjugated these dyes with a range of cytotoxic drugs with tumor homing properties and increased cytotoxic properties [14–18]. By integrating our cancer cell targeting, highly accumulating NIR dye with cytotoxic agent CIS, our group had previously developed a DZ-CIS conjugate that has shown significant efficacy on MYC-driven TP53 mutated CIS -resistant aggressive Burkitt’s lymphoma model [24]. In this study, we have demonstrated that RCC cells accumulate DZ-CIS in vitro and in vivo. DZ-CIS is very effective in cell killing of RCC cell lines while having no effect on normal kidney epithelial cells. Mouse tissue samples showed no evidence of kidney damage, as seen with CIS (Fig. 6B). We then found that DZ-CIS was highly effective when combined with mTOR inhibitors in RCC cells producing total cell killing in low micromolar ranges (Fig. 3). Our results suggest that the cytotoxic effect produced by DZ-CIS on RCC is mainly caused by apoptosis (Figs. 2A and B, and 6A). On the other hand, DZ-CIS may also affect the expression of critical genes to cause growth arrest or death. Our studies with Burkitt’s lymphoma, for instance, suggested that DZ-CIS might re-activate p53 and subsequently p21, with the potential of leading to cell cycle arrest and apoptosis. In p53 wild type RCC tumors p53 is suppressed, by various mechanisms like NF-κB increased expression [40] or p53 depletion through transglutaminase 2-chaperoned autophagy [41]. This mechanism however was not preserved in p53 mutated MYC driven Burkitt’s lymphoma model but presented with equivalent cytotoxic potency [24]. In the current study, RCC cells with wild type (Caki-1 and ACHN) or mutated p53 (786-O and SN12C) show similar sensitivity to DZ-CIS (Fig. 1), as DZ-CIS kills by attacking subcellular organelles, regardless of genetic makeup of the targets. The anti-proliferative properties of DZ-CIS are seen in both human RCC xenograft model and mouse RCC allograft model, suggesting that the effect is not dependent on hosts immune response. Treatment with DZ-CIS resulted in a significant decrease in SOX2 expression within tumor tissue, suggesting its effect on cells exhibiting stem cell or stem cell-like properties. There was also a significant decrease in the PECAM marker of angiogenesis, suggesting a possibility of enhancing the effects of anti-VEGF agents in RCC. Further molecular investigation is needed to fully elucidate the mechanism of DZ-CIS in RCC killing.

## Conclusions

Our study has indicated that DZ-CIS possesses bifunctional properties, exhibiting both tumor cell specificity and tumor cell killing activity. DZ-CIS kills kidney cancer cells with minimal renal toxicity and disregarding the intrinsic therapeutic resistance of the cancer cells. These unique features make DZ-CIS a promising antitumor agent for further preclinical investigation.

## Electronic supplementary material

Below is the link to the electronic supplementary material.

Supplementary Material 1

## Data Availability

The data that support the findings of this study are available from the corresponding author upon reasonable request.

## Abbreviations

RCC: Renal cell carcinoma
ccRCC: Clear cell renal cell carcinoma
HMCD: heptamethine carbocyanine dye
CIS: cisplatin
IC50: half maximal inhibitory concentration
VHL: von Hippel-Lindau
NIR: near-infrared
HPLC: high performance liquid chromatography
