# Supplementary figures and images for: A cisplatin conjugate with tumor cell specificity exhibits antitumor effects in renal cancer models
Source: BMC Cancer. 2023 Jun 2;23:499. doi: 10.1186/s12885-023-10878-3 (PMC10236852; doi:10.1186/s12885-023-10878-3)

Control

CIS ( $\mu$ M)

DZ-CIS ( $\mu$ M)

4

8

16

4

8

16

PARP

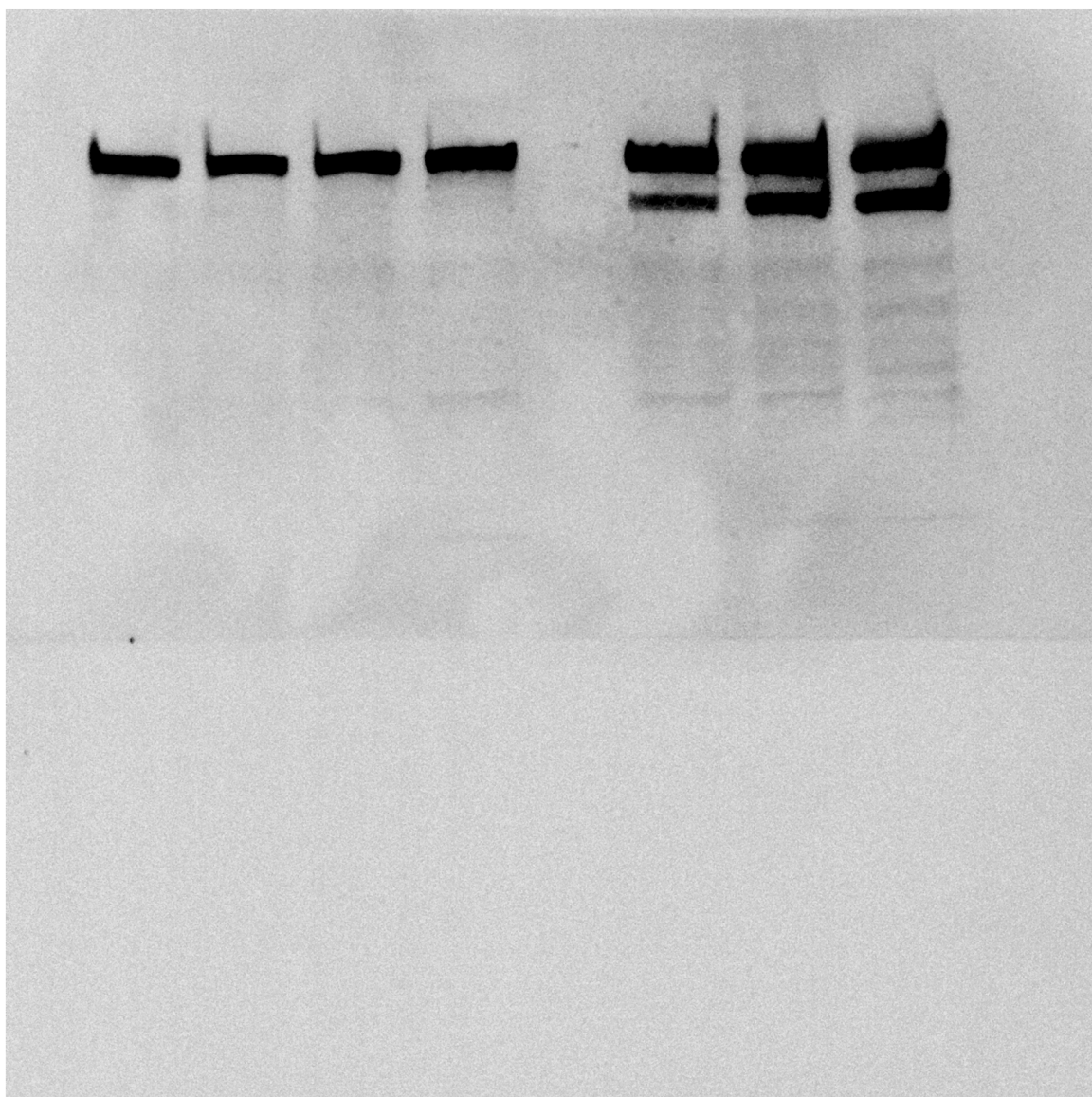

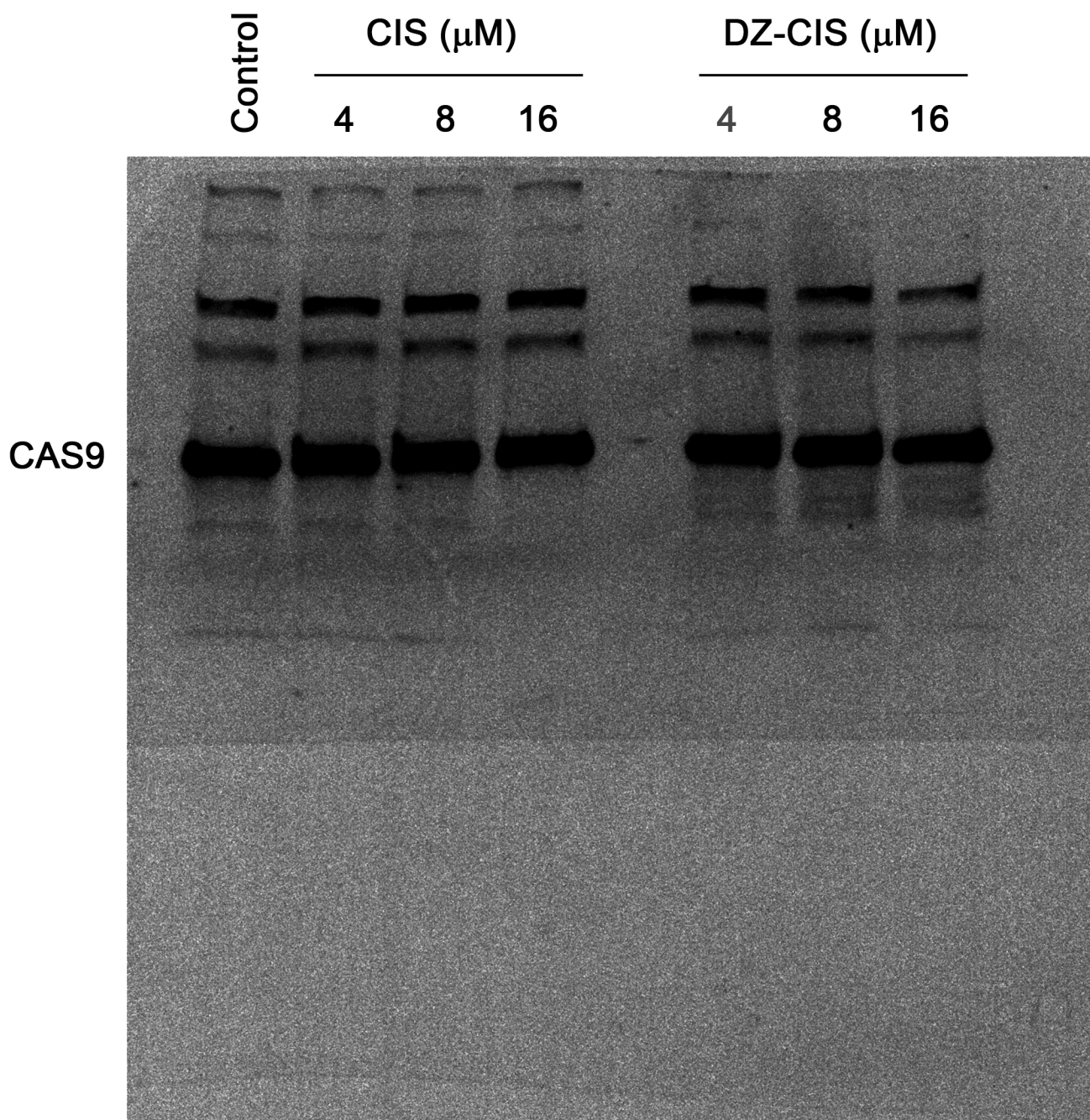

Control

CIS ( $\mu\text{M}$ )

DZ-CIS ( $\mu\text{M}$ )

4

8

16

4

8

16

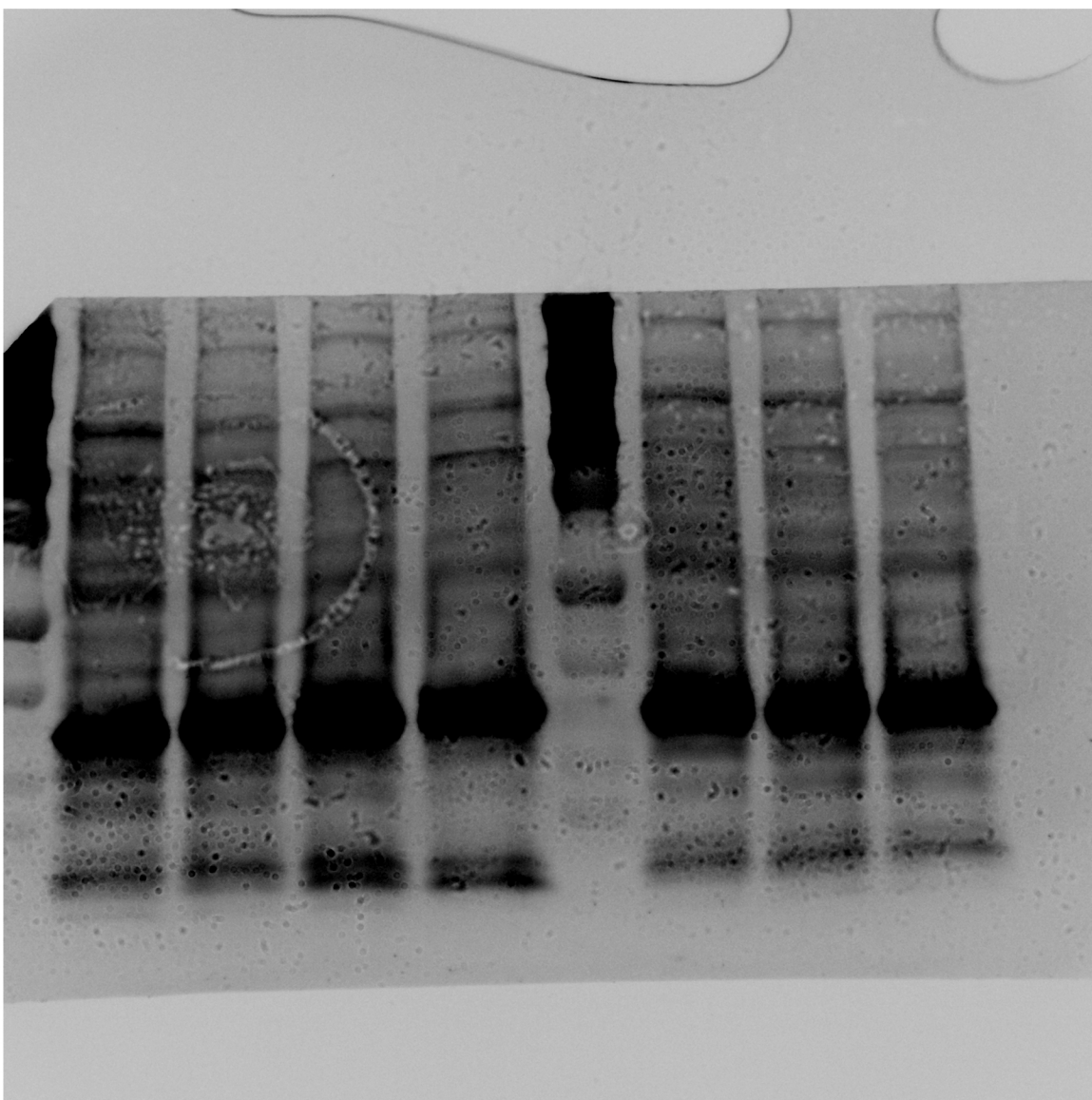

CAS3

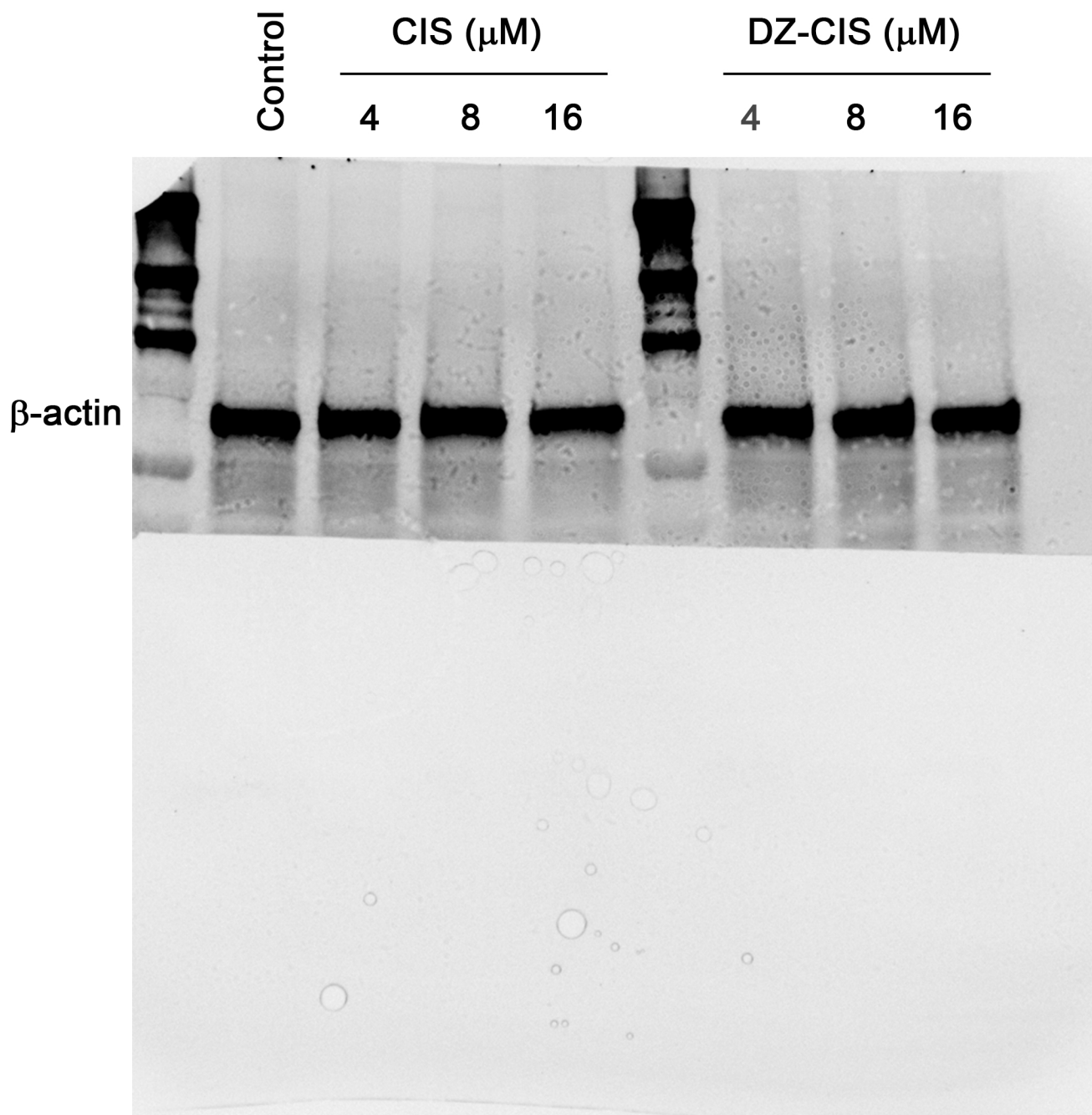

Supplement: Supplementary file 1 — Supplementary Material 1 [file 12885_2023_10878_MOESM1_ESM.pdf]
